# Evolution of pore characteristics and methane adsorption characteristics of Nanshan 1/3 coking coal under different stresses

**DOI:** 10.1038/s41598-022-07118-2

**Published:** 2022-02-24

**Authors:** Shuhao Fang, Hongqing Zhu, Min Gao, Xin He, Qi Liao, Lintao Hu

**Affiliations:** School of Emergency Management and Safety Engineering, China University of Mining and Technology-Beijing, Beijing, China

**Keywords:** Natural hazards, Solid Earth sciences, Coal, Macromolecules and clusters

## Abstract

To ascertain the evolution of pore characteristics and methane adsorption characteristics of the unit cell of Nanshan 1/3 coking coal under different stresses, proximate analysis, ultimate analysis, solid-state ^13^C nuclear magnetic resonance spectroscopy (^13^C-NMR) and X-ray photoelectron spectroscopy (XPS) experiments were performed on the coal samples, and a molecular unit cell model of 1/3 coking coal was established. As the increase of stress, pore diameter, proportion of larger pores, number of pores, surface area, and pore volume all decrease, the rate of decrease gradually decreases, and the smaller pores are less affected. Under 8 kinds of stress, the methane adsorption capacity and the overall system energies all conform to the Langmuir adsorption curve; as the stress increases, the methane adsorption capacity and the overall system energies both decrease, the rate of decrease gradually decreases, and the order of the adsorbed methane increases. Stress changes the methane adsorption capacity by changing the pore characteristics of the unit cell, and the stress has a more obvious effect on larger pores. As the stress increases, the speed of the stress's influence on the pores weakens. This has certain guiding significance for studying the saturated adsorption capacity of methane under different original in-situ stresses.

## Introduction

Coal is a complex and heterogeneous polymeric material composed of aromatic, aliphatic, various functional groups and microcrystalline graphite sheet^1–4^. Coal has the properties of porous media, divided into micropores (< 2 nm), mesopores (2–50 nm) and macropores (> 50 nm)^5–7^. The adsorption of methane is mainly in micropores and mesopores^8,9^. There are many ways to calculate the pore information of a material.

The Poreblazer program can obtain information about the pore structure, but it also has some shortcomings^10^. For example, when the pore size is smaller than the size of the N_2_ molecule, the pore size distribution (PSD) of the material cannot be displayed^11^. The Zeo +  + program is based on Voronoi decomposition, which can better characterize the pore structure properties^12,13^. The radica Voronoi tessellation can effectively calculate the pore structure of systems containing atoms of different radii^14^. Martin et al.^15^ proposed the similarity coefficient based on the void space structure of the material through Voronoi hologram. Martin et al.^16^ proposed an algorithm for the assembling of crystalline porous polymer structure models that can be used in Zeo +  + software. PSD characterize the proportion of different pore sizes in the research object^17^. Brochard et al.^18^ concluded that the poromechanical response of a microporous medium to adsorption significantly depends on the PSD.

The radial distribution function (RDF) is the ratio of the local density in the radial to the average bulk density of the system, which can reflect the characteristics of particle aggregation and order and structure information^19–22^.

The gas adsorption theory of coal includes the Langmuir model of single-layer adsorption proposed by Langmuir in 1916^23,24^, the BET model of multilayer adsorption proposed by Brunauer, Emmett and Teller^25^ in 1938, the DS model proposed by Dubinin and Stoeckli^26^ in 1980, and the adsorption potential model^27^. etc. A large amount of literature has studied the influencing factors of coal's methane adsorption capacity. Zhang et al.^28^ concluded that temperature affects the structure of coal molecules, thereby affecting gas adsorption. Liu et al.^29^ concluded that the micropores of coal play an important role in gas adsorption capacity. Liu et al.^30^ found that the adsorption of methane in coal is mainly micropore filling adsorption. Yan and Yin et al.^31,32^ believe that the pore structure and specific surface area of coal directly affect the methane adsorption capacity. Bai et al.^33^ concluded that the injection of N_2_ and CO_2_ into coal inhibits methane adsorption. Wen et al.^34^ concluded that temperature inhibit coal gas adsorption. Zhang et al.^35^ studied the influence of micropore parameters and oxygenic functional groups on the adsorption capacity of methane. Wang and Pan et al.^36,37^ studied the influence of temperature, pressure and moisture content on the adsorption capacity of methane. Mosher, Liu and Wang et al.^38–40^ studied the effect of pore size and pressure on the methane adsorption capacity of coal. Nie, Chen, Zhao and Liu et al.^41–44^ studied the influence of pore structure characteristics on coal's gas adsorption capacity through simulation and experiment. Shan and Yeganegi et al.^45,46^ studied the influence of pore shape on the gas adsorption capacity of coal. Liu et al.^47^ studied the influence of pressure and moisture content on the gas adsorption capacity of coal. Dang et al.^48^ studied the influence of functional groups, pressure and electrostatic on the gas adsorption capacity of coal.

Based on the above literature analysis, the research on methane adsorption characteristics of coal seam under three-dimensional stress is insufficient. Based on the experiment, the molecular model of Nanshan 1/3 coking coal is established, this article comprehensively analyzes the evolution of the pore characteristic parameters of the unit cell model and the adsorption characteristics of methane under different stress conditions (the six sides of the unit cell are subjected to the same stress).The research has a certain guiding significance for the evolution of coal pore characteristics and the saturated adsorption capacity of methane under different in-situ stress conditions.

## Coking coal molecular model construction

### Coal sample test results

Proximate analysis, ultimate analysis, ^13^C-NMR and XPS tests were performed on Nanshan 1/3 coking coal. The methods refer to literature^49–51^. The results of proximate analysis and ultimate analysis are shown in Table 1.

**Table 1:** Proximate and ultimate analyses of Nanshan 1/3 coking coal.

Coal sample	R^o^_max_	Proximate analysis ω/%			Ultimate analysis ω_daf_/%					Sample coal type
		*M*_ad_	*A*_d_	*V*_daf_	C	H	O_diff_	N	S_t,d_	
Nanshan	1.56	1.17	18.43	32.31	76.84	4.88	17.11	0.78	0.39	1/3 J

R^o^_max,_ the vitrinite reflectance values; (see the literature^52^ for the meaning of symbols).

**Figure 1:**
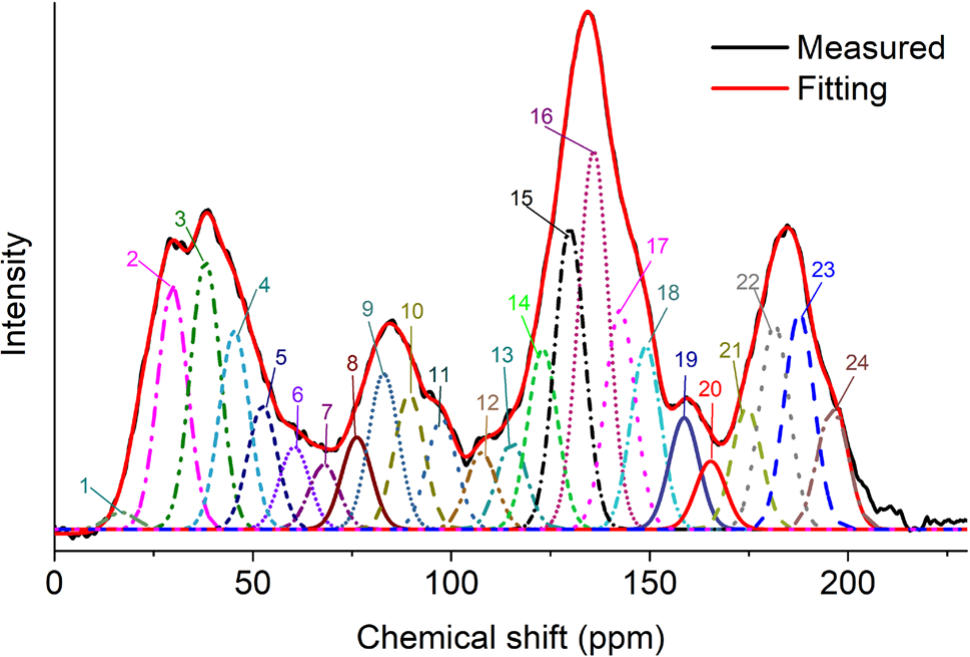
^13^C-NMR spectrum split peak fitting.

For the peak fitting of the ^13^C-NMR spectrum, the structure attribution of the carbon element is judged according to the chemical shift value^52–57^, and the peak fitting is shown in Fig. 1. According to the relative area value of each structure, the ^13^C-NMR structural parameters of the coal sample were calculated, as shown in Table 2.

**Table 2:** ^13^C-NMR structural parameters in coking coal (See the literature^58^ for the meaning of symbols).

Type	*f*_*a*_	*f*_*a*_^*C*^	*f*_*a*_^*'*^	*f*_*a*_^*N*^	*f*_*a*_^*H*^	*f*_*a*_^*P*^	*f*_*a*_^*S*^	*f*_*a*_^*B*^	*f*_*al*_	*f*_al_*	*f*_*al*_^*H*^	*f*_*al*_^*O*^
1/3 coking	60.39	19.86	40.53	23.51	17.02	7.76	5.78	9.97	39.61	12.71	9.7	17.2

According to the structure parameter value, calculate the ratio of aromatic bridge carbon to perimeter carbon, X_BP_, as shown in Eq. (1)^58^.

1 $${X}_{BP}=\frac{{f}_{a}^{B}}{{f}_{a}^{H}+{f}_{a}^{P}+{f}_{a}^{S}}=0.326$$

**Figure 2:**
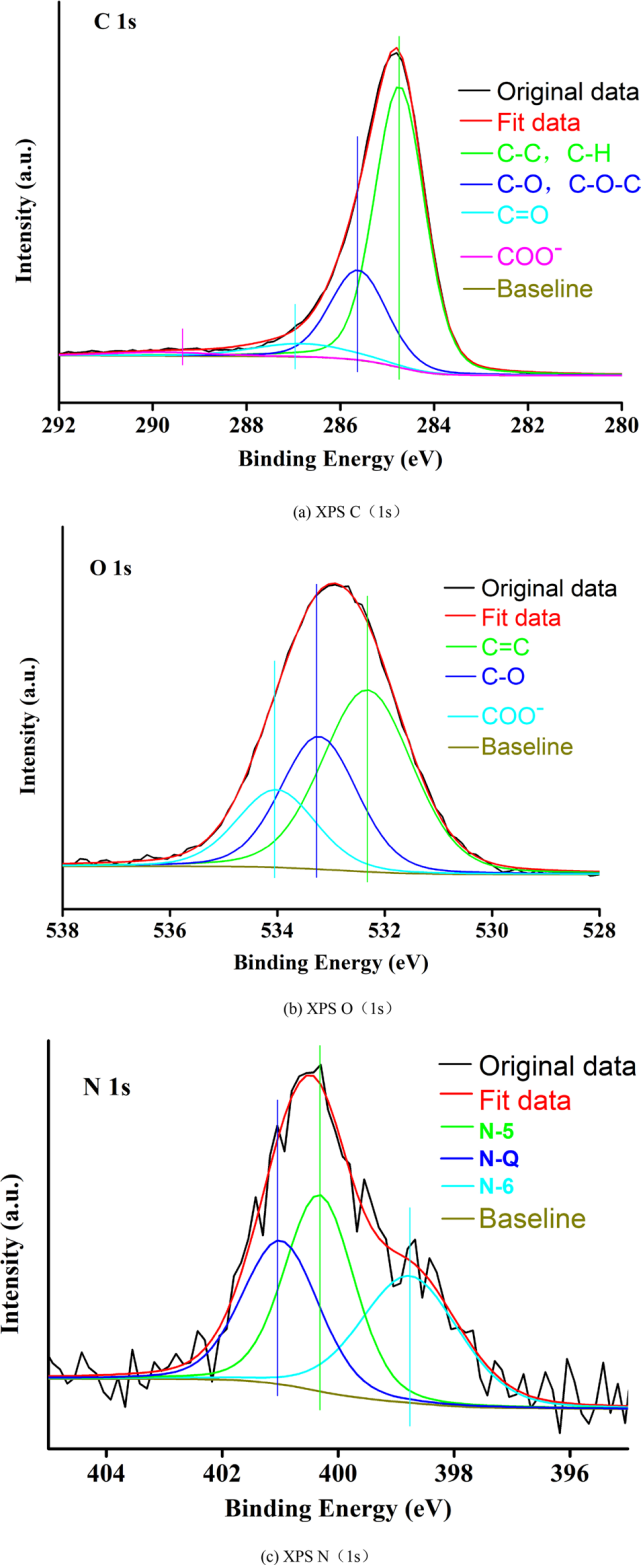
The split peak spectra.

The Avantage software was used to perform peak fitting of heteroatoms (C, O, N) in XPS^59^, ignoring the lower content of S^60^, and judge the structure attribution of C, O, N elements based on chemical shifts^61–63^. The C(1s), O(1s) and N(1s) split peak spectra of 1/3 coking coal are shown in Fig. 2a–c. The proportion of the structure is calculated by the relative area, and the structure parameters of each peak position shown in Table 3.

**Table 3:** Structure attribution of C, O and N element.

Element	Peak	Area (P)	Proportion (%)	Types
C	284.74	43,654.13	67.37	C–C, C–H
	285.62	15,942.67	24.61	C–O, C–O–C
	287.01	4057.39	6.26	C=O
	287.76	1139.31	1.76	COO–
O	532.32	13,165.86	50.07	C=O
	533.32	8180.55	31.11	C–O
	534.02	4949.69	18.82	COO–
N	398.77	400.26	33.74	Pyridinic nitrogen (N-6)
	400.32	433.74	36.56	Pyrrolic nitrogen (N-5)
	401.01	352.31	29.70	Quaternary nitrogen

### Unit cell model construction

Based on the results of proximate analysis and ultimate analysis, different aromatic hydrocarbon have different X_BP_ values and XPS analysis results^64^, it is concluded that the molecule contains three naphthalene, one phenanthrene, one chrysene, and one pyridinic nitrogen, the number of oxygen atoms is 30, including 24 oxygen-containing functional groups, of which 10 carbonyl oxygens, 6 carboxyl oxygens, and 8 ether bond oxygens.

**Figure 3:**
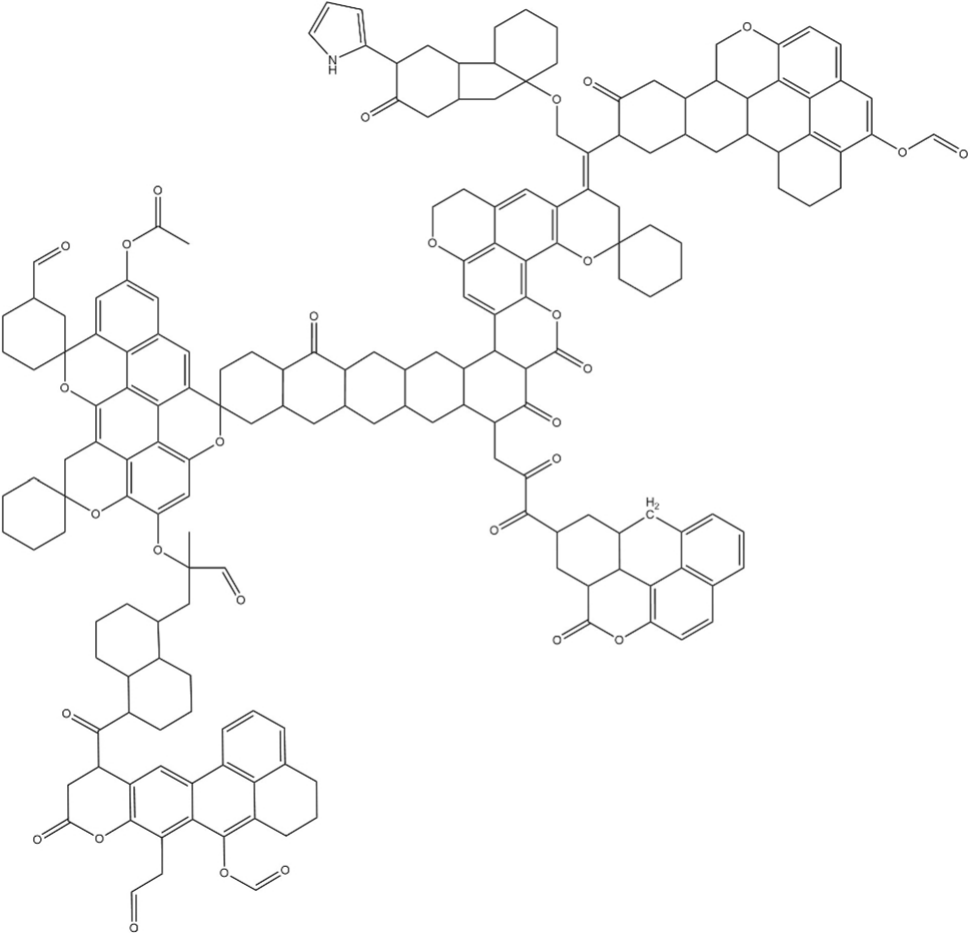
Molecular structure model diagram of Nanshan 1/3 coking coal.

The calculated X_BP_ value is 0.296, and the final molecular formula is determined to be C_181_H_179_NO_30_. Using ChemDraw software, the analyzed molecular structure is combined and constructed, and the macromolecular structure of coking coal is shown in Fig. 3.

**Figure 4:**
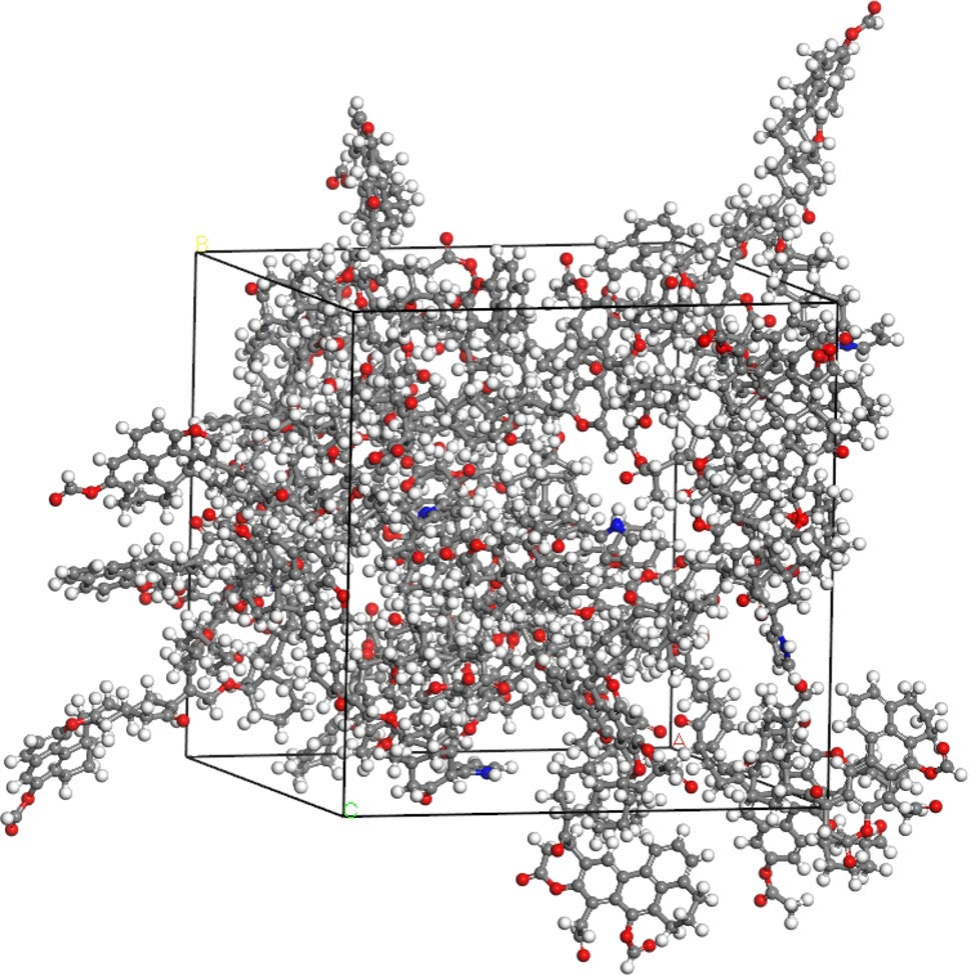
Unit cell model.

The molecular model was geometrically optimized and annealed in the Forcite module of Materials Studio software, and the temperature was set at 293.15 K. Based on the optimized coal molecules, a unit cell containing 7 coal molecules is established. After geometric optimization and annealing, the model with the lowest energy is selected, as shown in Fig. 4. The lattice parameters are a = b = c = 30.7748 Å and α = β = γ = 90°.

## Pore characteristics of the unit cell model

For the established lowest energy unit cell model, the same stress is applied to all six faces of the unit cell. The stress is 0 GPa, 0.2 GPa, 0.4 GPa, 0.6 GPa, 0.8 GPa, 1 GPa, 1.2 GPa and 1.4 GPa, a total of 8 conditions. Then the molecular models were geometrically optimized, annealed and dynamically balanced, and the respective unit cell models were finally determined.

**Figure 5:**
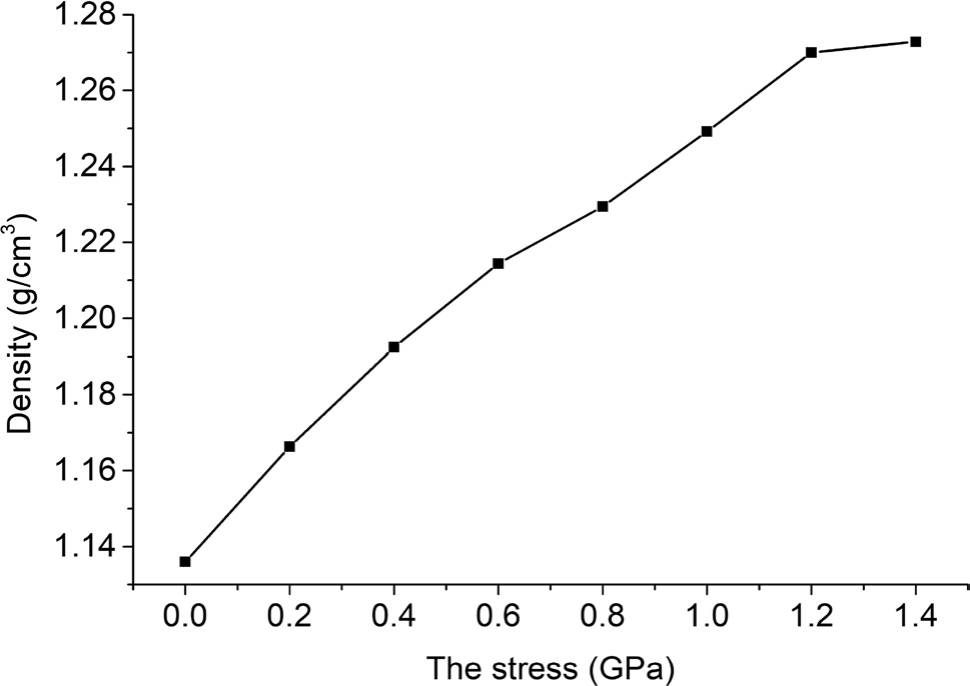
Relationship between stress and density.

The density of the unit cell model under 8 kinds of stress is shown in Fig. 5. As the stress increases, the density of the unit cell gradually increases, and the rate of increase gradually decreases.

**Figure 6:**
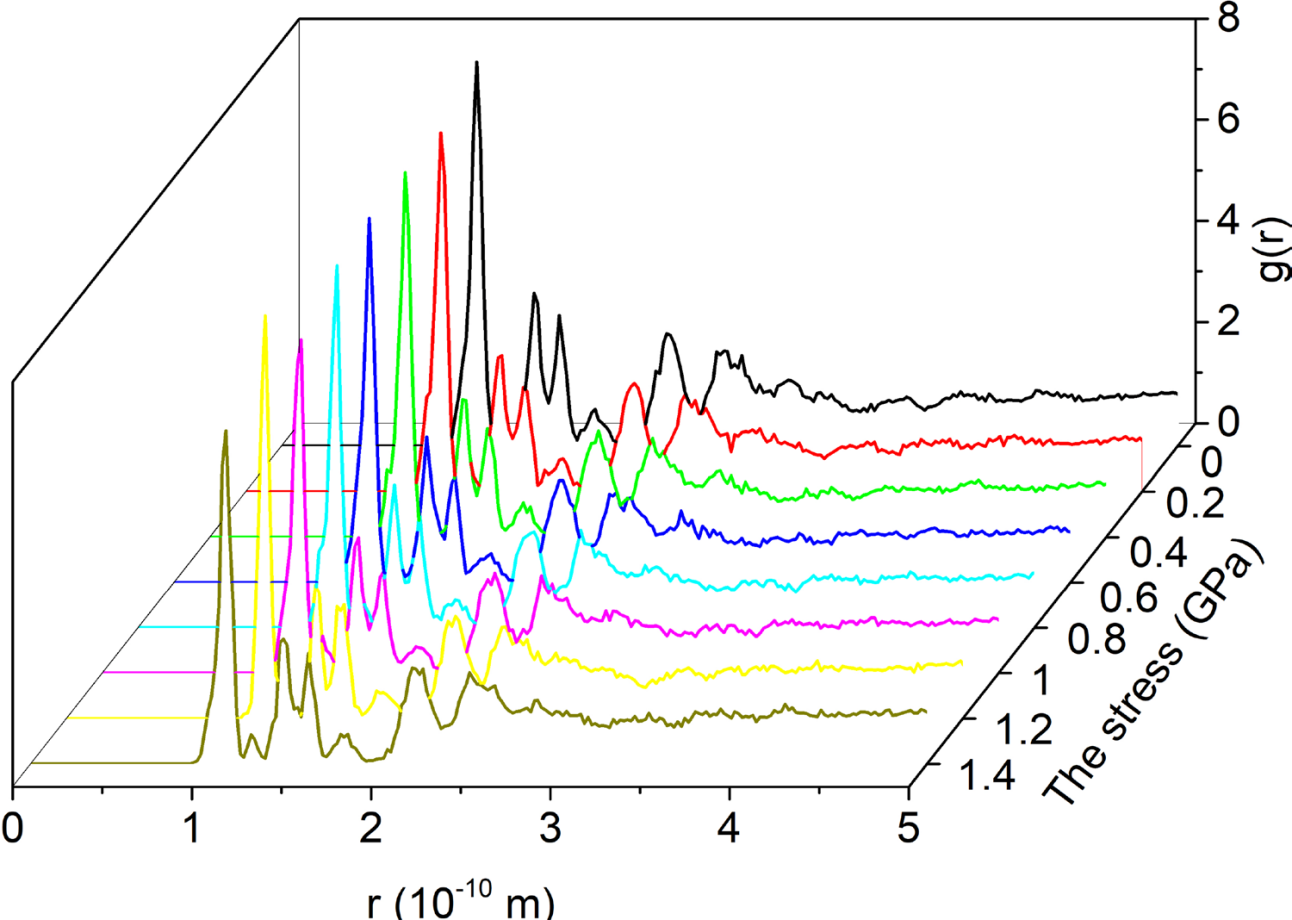
RDF of unit cell.

The RDF of the unit cell model under 8 kinds of stress is shown in Fig. 6. Under different stresses, the RDF of the unit cell is basically the same, indicating that the order of the unit cell is basically unchanged.

Use the zeo +  + -0.3 code to calculate the pore size, PSD, constrained stochastic ray tracing, surface area and pore volume of the unit cell under 8 kinds of stress. In order to find out the characteristics of the pores, the Monte Carlo algorithm is used, the radius of the probe is 0.5 Å, and the sample is 100,000.

### Pore size

**Figure 7:**
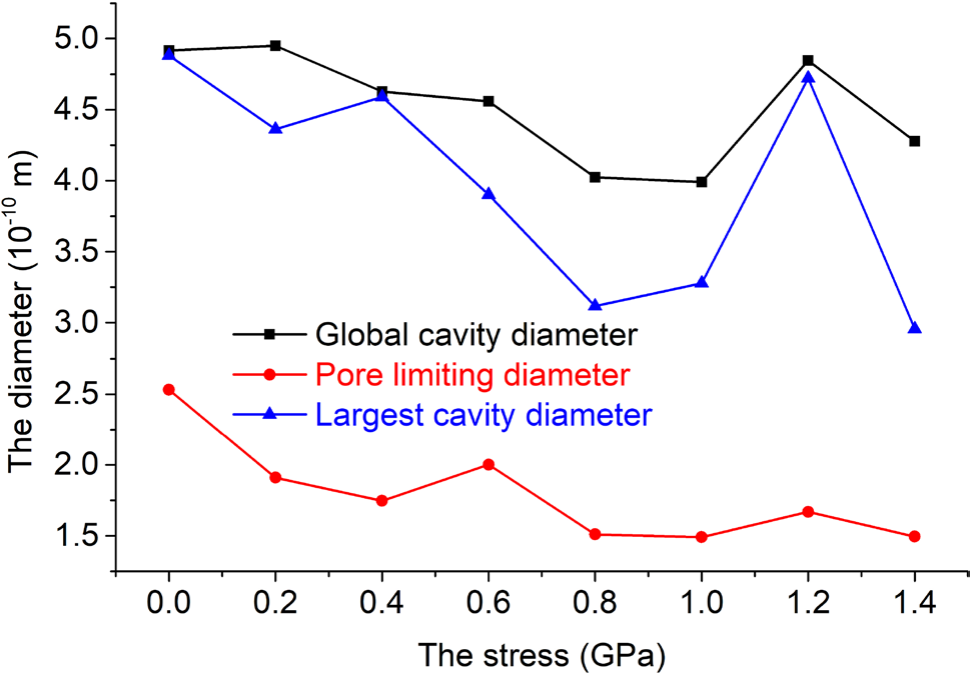
Relationship between stress and pore diameter.

The pore size of the unit cell model under 8 kinds of stress is shown in Fig. 7. The three pore sizes are: global cavity diameter > largest cavity diameter > pore limiting diameter. As the stress increases, the global cavity diameter, pore limiting diameter, and largest cavity diameter all show a smaller decreasing trend.

**Figure 8:**
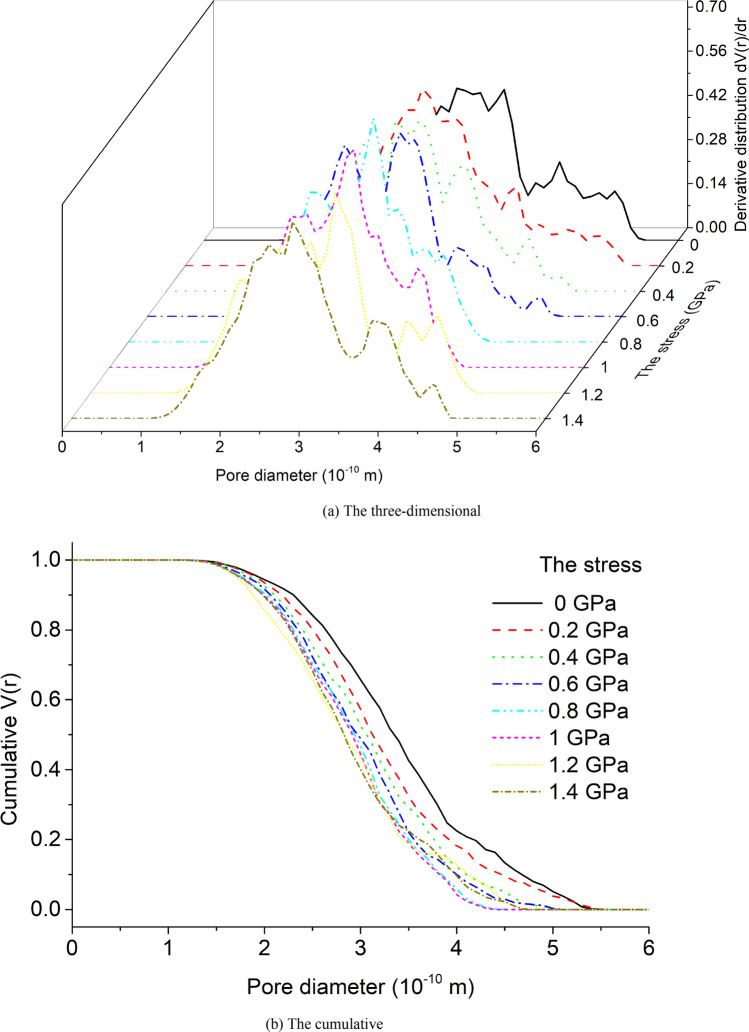
PSD of the unit cell model under eight kinds of stress.

The three-dimensional and cumulative PSD of the unit cell model under eight kinds of stress are shown in Fig. 8a,b. The unit cell model under 8 kinds of stress shows a large peak when the pore diameter is smaller, and a small peak when the pore diameter is larger, which indicates that the proportion of smaller pores is larger than that of larger pores. As the stress increases, the maximum pore size of the unit cell model decreases, and the range of PSD decreases, showing a trend of increasing the proportion of smaller pores and decreasing the proportion of larger pores, and the trend is gradually decreasing.

**Figure 9:**
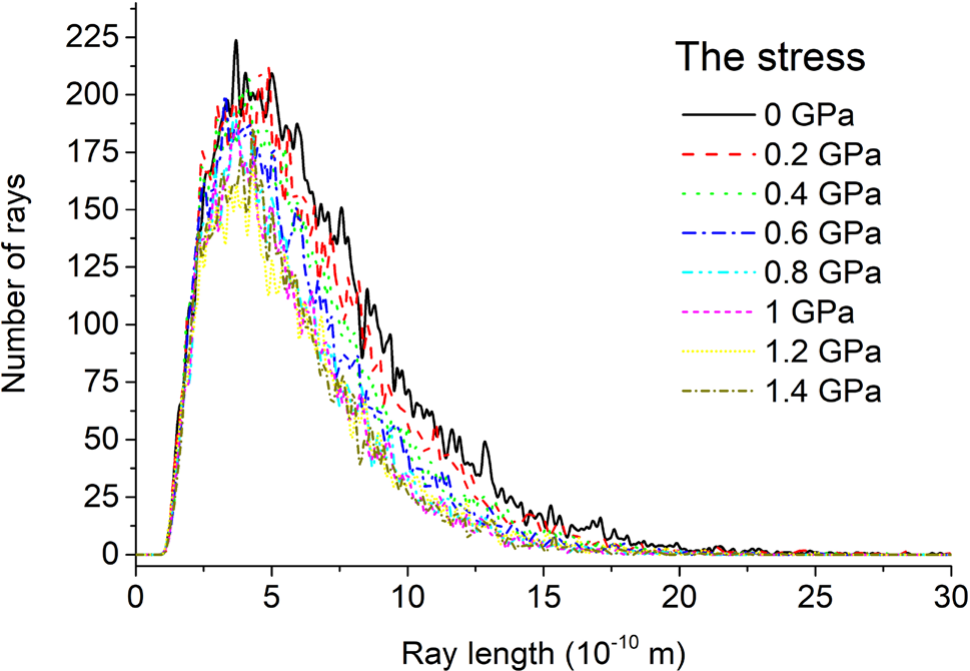
Constrained stochastic ray trace distribution of unit cell model under 8 kinds of stress.

The PSD and constrained stochastic ray tracing have distinct but complementary characteristics when detecting pores^65^. The constrained stochastic ray tracing distribution of the unit cell model under 8 kinds of stress is shown in Fig. 9. The constraint stochastic ray tracing distributions of the unit cell models under the eight stress conditions are all asymmetric single peaks. The right side of the peak is much smoother than the left side, and the ray lengths corresponding to the peaks are basically the same. After being greater than the peak ray length, as the ray length increases, the number of the same ray length decreases. As the stress increases, the number of rays with the same length decreases, and the speed at which the number of rays decreases gradually decreases; the number of rays with a smaller length is basically unchanged. This shows that as the stress increases, the number of all pores in the unit cell model decreases, and the rate of decrease gradually decreases, and the smaller pores are less affected.

### Surface area and volume

**Figure 10:**
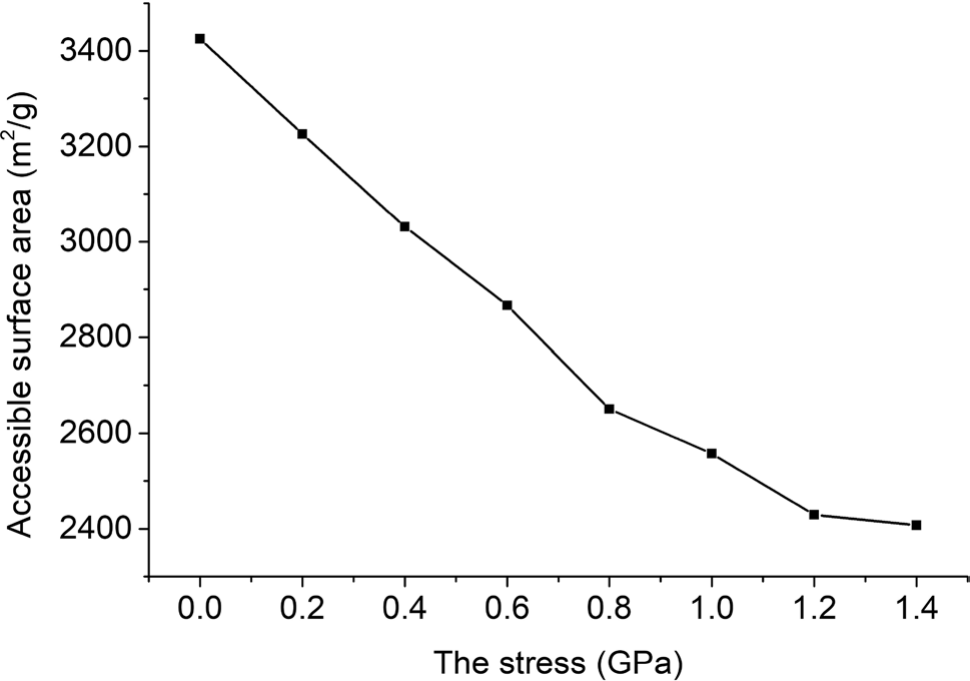
Relationship between stress and accessible surface area.

The accessible surface area is more suitable for characterizing adsorbed porous solids than the Connolly surface area^66^. The accessible surface area depends on the size of the probe molecule. The accessible surface area of the unit cell model under 8 kinds of stress is shown in Fig. 10. As the stress increases, the accessible surface area gradually decreases, and the rate of decrease gradually decreases.

The accessible and probe-occupiable pore volume is the closest to the experimentally measured pore volume. The geometric pore volume is the upper limit of this value, while the accessible pore volume greatly underestimates the experimental value^67^.

**Figure 11:**
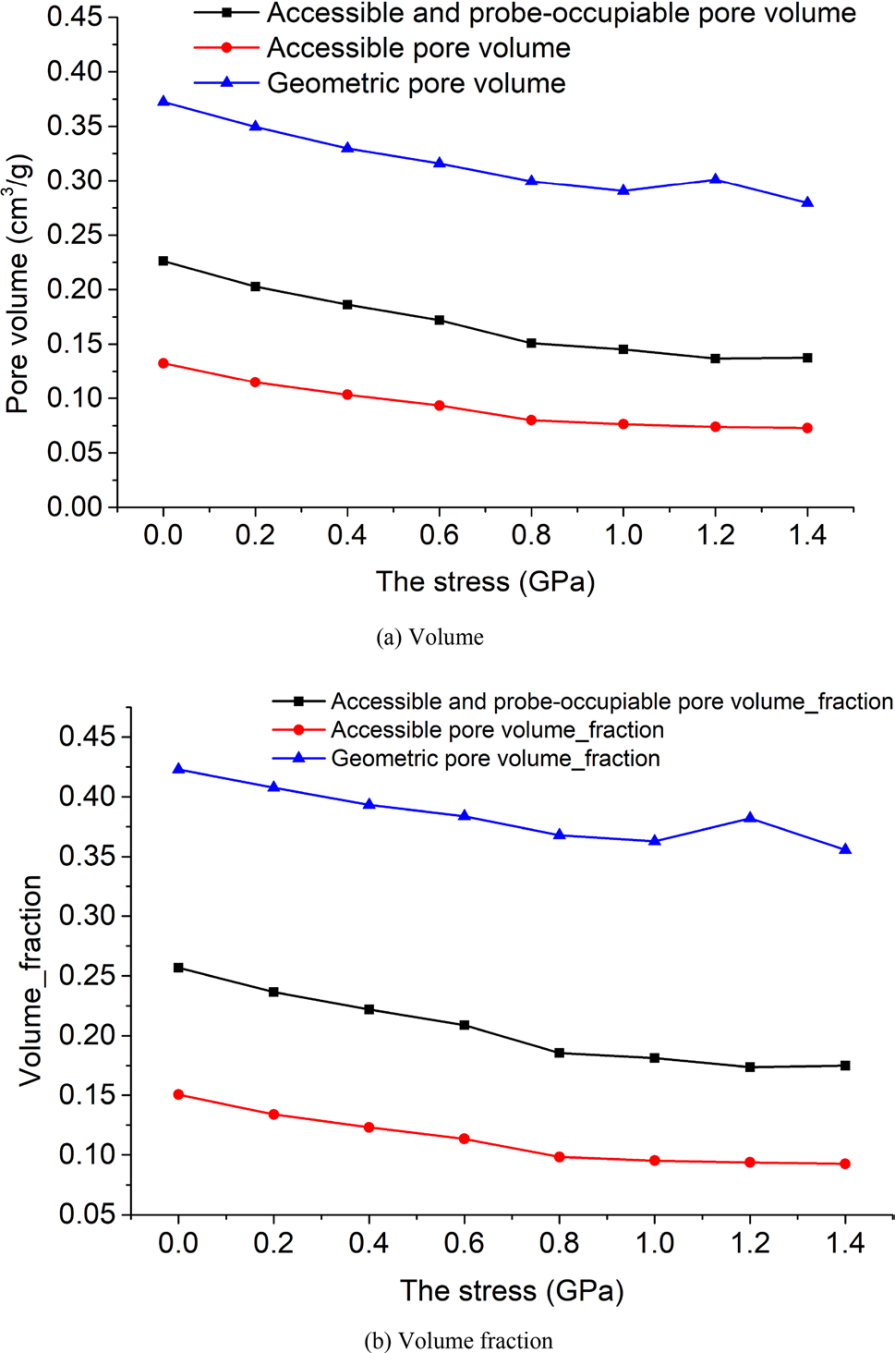
The pore volume of the unit cell model under 8 kinds of stress.

The pore volume and volume fraction of the unit cell model under 8 kinds of stress are shown in Fig. 11a,b. The relationship between the three pore volumes is consistent with the theory. As the stress increases, the values of accessible and probe-occupiable pore volume, geometric pore volume and accessible pore volume generally show a decreasing trend. The volume fraction of accessible and probe-occupiable pore volume is approximately 20%. The volume fraction of geometric pore volume is about 40%, which is twice of the volume fraction of accessible and probe-occupiable pore volume. The volume fraction of accessible pore volume is about 10%, which is half of the volume fraction of accessible and probe-occupiable pore volume.

## Molecular dynamics simulation

### Fugacity

The Soave–Redlich–Kwong equation can be expressed by the Eq. (2).^68^

2 $${Z}^{3}-{Z}^{2}-\left({B}^{2}+B-A\right)Z-AB=0$$

where Z is compression factor, $A=a\alpha \left(T\right)p/{R}^{2}{T}^{2}$, $\mathrm{B}=bp/RT$, $\mathrm{a}=0.45724{R}^{2}{T}_{c}^{2}/{p}_{c}$, $b=0.08664R{T}_{c}/{p}_{c}$, $\mathrm{\alpha }\left(T\right)={\left[1+k\left(1-{T}_{r}^{0.5}\right)\right]}^{2}$, $k=0.48+1.574\omega -0.176{\omega }^{2}$, ${T}_{r}=T/{T}_{c}$, $\omega$ is the acentric factor of methane, 0.008, ${T}_{r}$ is the contrast temperature , K, T is the temperature, 293.15 K, ${T}_{c}$ is the critical temperature of methane, 190.56 K, p is the pressure, MPa, ${p}_{c}$ is the critical pressure of methane, 4.5992 MPa, R is molar gas constant, 8.31 J/(mol*K).

The fugacity coefficient of methane ($\varphi$) is shown in Eq. (3).

3 $$\mathit{ln}\varphi =Z-1-\mathit{ln}\left(Z-B\right)-\frac{A}{B}\mathit{ln}\frac{Z+B}{Z}$$

The fugacity of methane ($f$) is shown in Eq. (4).

4 $$f=\varphi p$$

**Figure 12:**
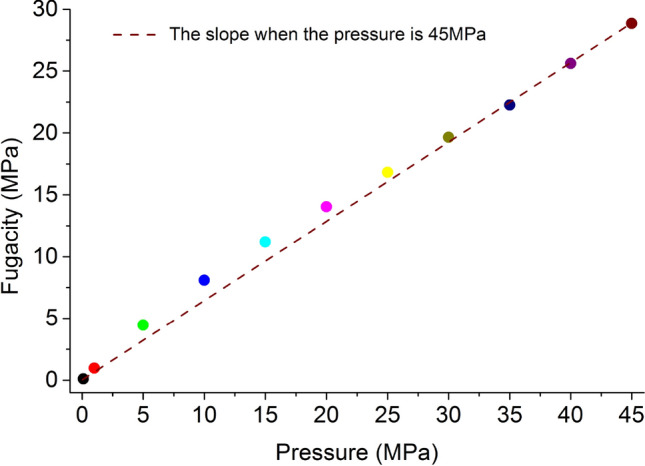
The relationship between the fugacity of methane and pressure.

The corresponding relationship between pressure and fugacity calculated by Eqs. (2), (3) and (4) is shown in Fig. 12. The fugacity coefficient of methane is less than 1, and decreases with the increase of pressure, and finally stabilizes.

The unit adsorption capacity of methane is equal to the ratio of the number of adsorbed methane to the molar mass of unit cell, that is, Eq. (5).

5 $$x=\frac{{N}_{\mathrm{CH}4}}{{\mathrm{M}}_{M}*{\mathrm{N}}_{\mathrm{M}}}$$

where *N*_*CH4*_ is the number of adsorbed methane, *N*_*M*_ is the number of coal molecules, 7, *M*_*M*_ is the molar mass of coal molecules, 2.845 kg/mol, *x* is the amount of adsorbed methane, mol/kg.

### Methane adsorption results

**Figure 13:**
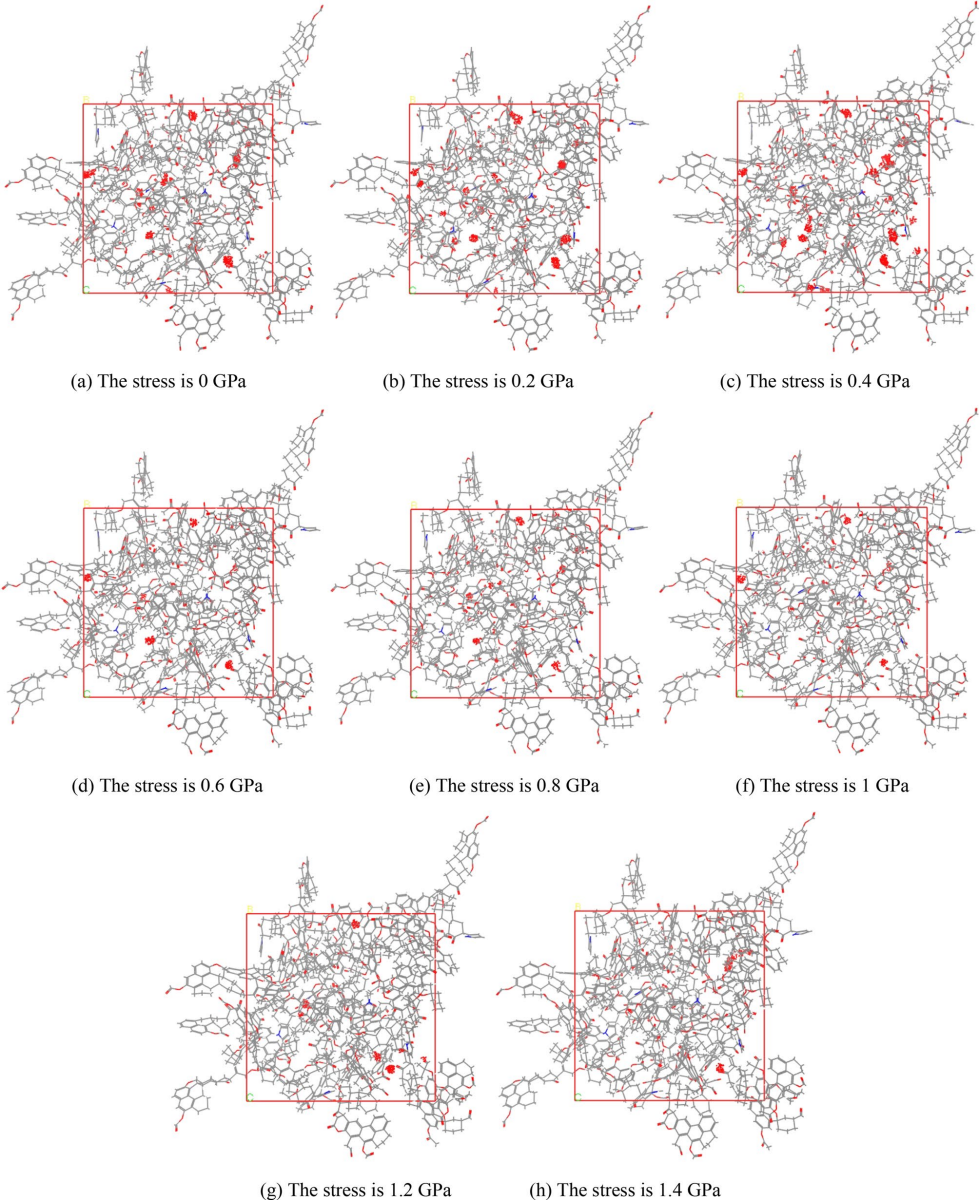
The adsorption results of unit cells when the methane injection pressure is 45 MPa.

The simulation was carried out using the “Sorption in the Forcite” module of the Materials Studio. In the simulation, the injected pressure of methane is 0.1 MPa, 1 MPa, 5 MPa, 10 MPa, 15 MPa, 20 MPa, 25 MPa, 30 MPa, 35 MPa, 40 MPa, 45 MPa, a total of 11 conditions. Under the action of different stresses, the adsorption results of unit cells when the methane injection pressure is 45 MPa are shown in Fig. 13a–h.

**Figure 14:**
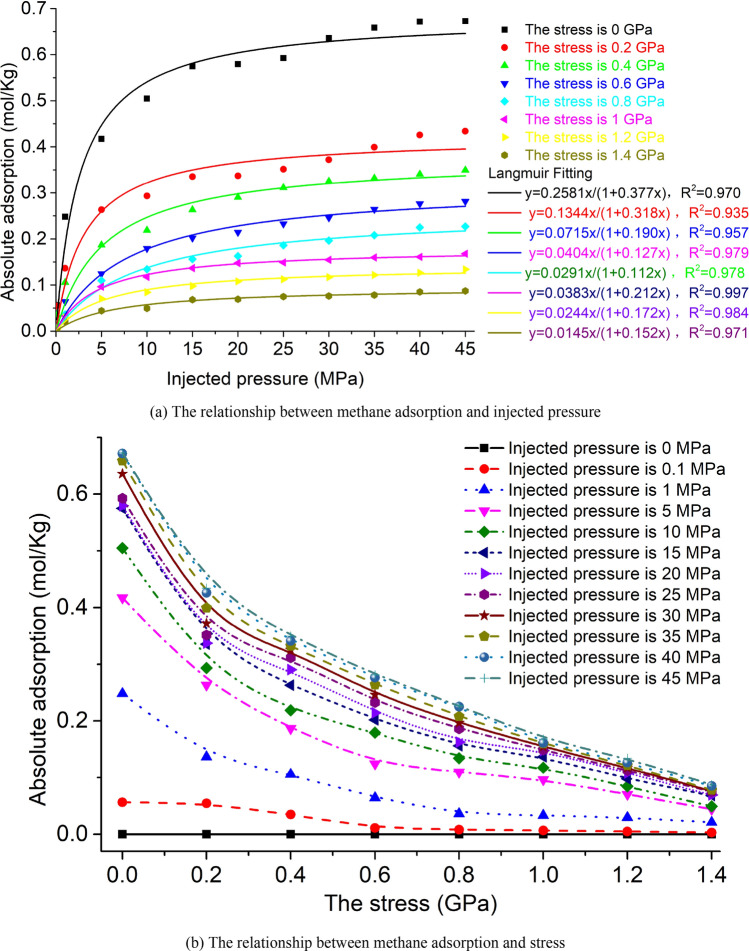
Methane adsorption capacity under different stress and injected pressure conditions.

The methane injected pressure is converted to the corresponding fugacity application according to Fig. 12, and the amount of methane adsorbed by the simulation is converted according to Eq. (4). The adsorption capacity of the unit cell under the action of 8 kinds of stress under different methane injected pressure conditions is shown in Fig. 14a,b.

Langmuir fitting was performed on the methane adsorption capacity under 8 kinds of stress, as shown in Fig. 14a. The correlation coefficients of the Langmuir fitting under 8 kinds of stress are all greater than 0.93, indicating that the methane adsorption capacity conforms to the Langmuir adsorption curve. The amount of methane adsorption showed a Langmuir curve increase with the increase of methane injection pressure. Under different stresses and the same methane injection pressure, the amount of methane adsorption decreases as the stress increases. The higher the methane injection pressure, the faster the amount of methane adsorption decreases with the increase of stress.

**Figure 15:**
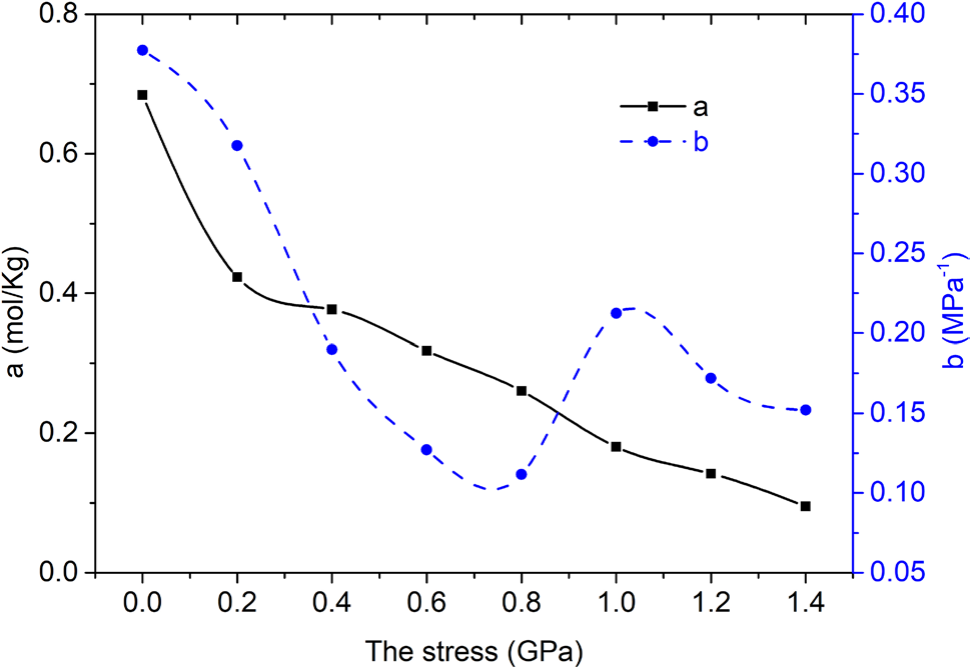
The relationship between stress and a, b.

Take stress as the abscissa and Langmuir adsorption constants a and b as the ordinate respectively to draw curves, as shown in Fig. 15.

With the increase of stress, the value of the limit adsorption capacity a of methane decreases, and the value of the adsorption constant b first decreases rapidly and then increases.

### Overall system energies and RDF of methane

**Figure 16:**
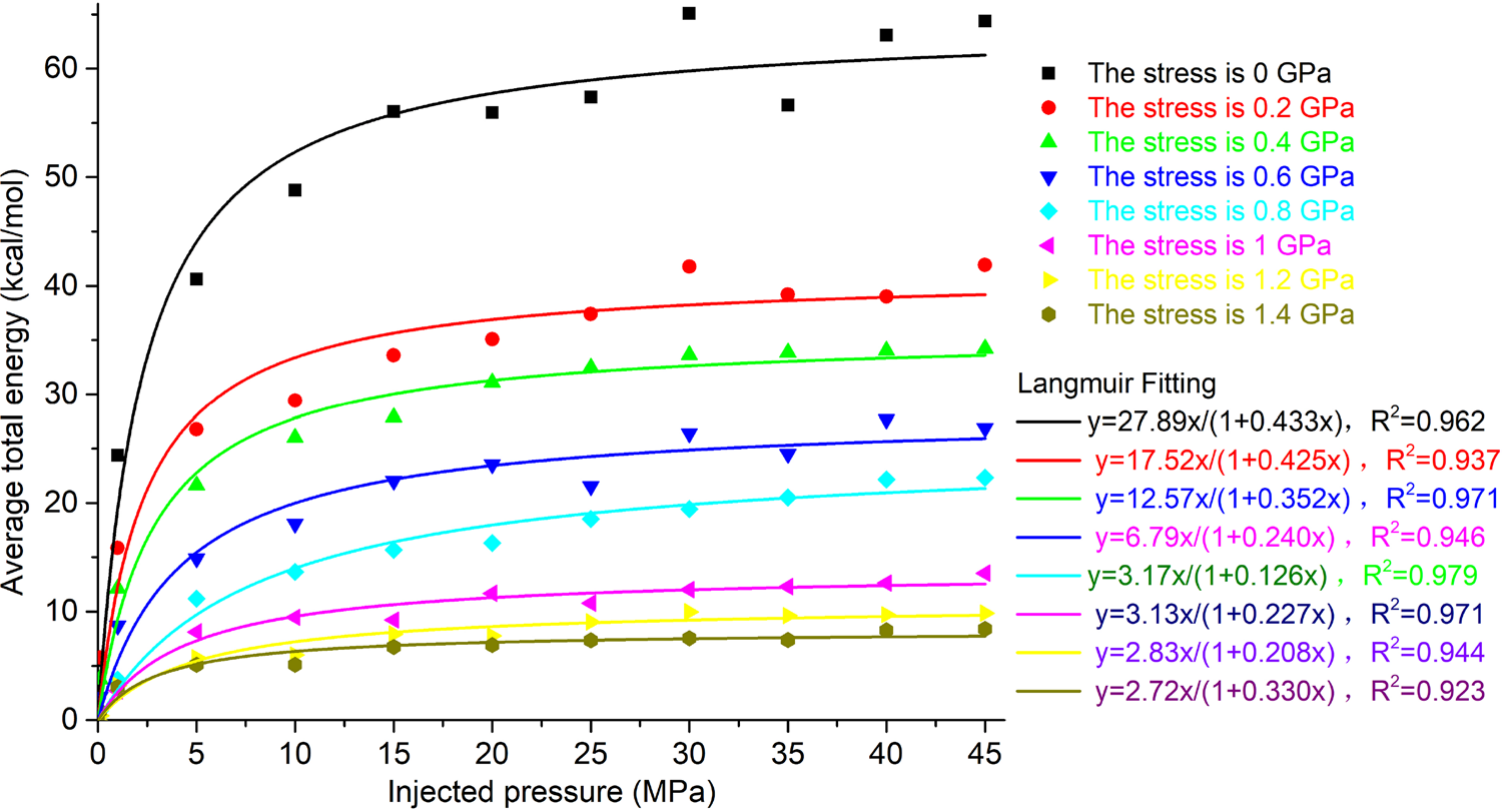
Overall system energies under different stress and injection pressure conditions.

The overall system energies of the unit cell under 8 kinds of stresses under different methane injection pressure conditions is shown in Fig. 16. Langmuir fitting was performed on the overall system energies under eight kinds of stress conditions. The correlation coefficients of Langmuir fitting under the eight kinds of stress were all greater than 0.92, indicating that the overall system energies conformed to the Langmuir curve. As the stress increases, the overall system energies decrease, and as the injection pressure increases, the overall system energies increase, and the change of the overall system energies is basically the same as the change in the amount of methane adsorption.

**Figure 17:**
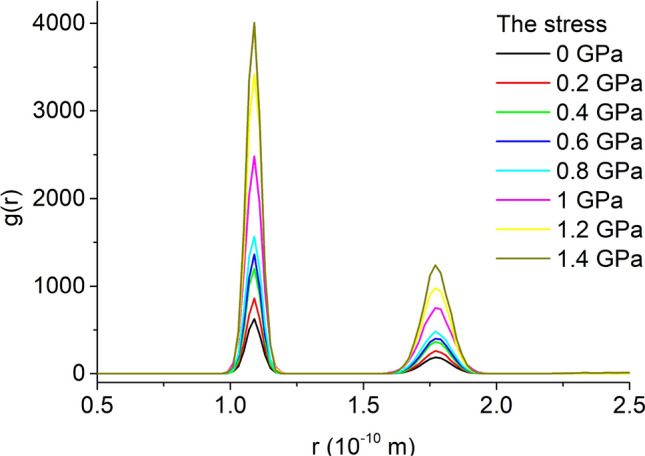
Radial distribution function of methane.

Select the unit cell model at 45 MPa methane injection pressure under 8 kinds of stresses to calculate the RDF of methane, as shown in Fig. 17. There are two peaks in the RDF of methane, the closer peak is higher. As the stress increases, the peak value also increases, indicating that the greater the stress, the greater the order of the adsorbed methane. The greater the stress, the smaller the amount of methane adsorbed, and its order will increase, which is consistent with the RDF of methane.

## Conclusions

The application of stress causes the pores of the unit cell to shrink, and the larger pores are reduced by a higher rate, which leads to a decrease in the methane adsorption capacity of the unit cell. The change in applied stress and the pore shrinkage of the unit cell are not linear.

1. The unit cell models under 8 kinds of stresses of 0 GPa, 0.2 GPa, 0.4 GPa, 0.6 GPa, 0.8 GPa, 1 GPa, 1.2 GPa, and 1.4 GPa were established. As the stress increases, the density of the unit cell gradually increases, and the rate of increase decreases, and the order of the unit cell remains unchanged.
2. With the increase of stress, the global cavity diameter, pore limiting diameter, and largest cavity diameter all decrease, the proportion of smaller pores increases, the proportion of larger pores decreases, and the number of pores decreases, the speed of reduction gradually decreases, and the smaller pores are less affected. As the stress increases, the accessible surface area, accessible and probe-occupiable pore volume, geometric pore volume and accessible pore volume all show a decreasing trend.
3. The fugacity coefficient of methane is less than 1, and decreases with the increase of pressure, and finally stabilizes. Under 8 kinds of stresses, the amount of methane adsorption conforms to the Langmuir adsorption curve. As the stress increases, the methane adsorption performance of the unit cell decreases, and the rate of decrease gradually decreases. The change of overall system energies is basically the same as the change of amount of methane adsorption. As the stress increases, the amount of methane adsorbed decreases, and the order of methane increases.

## Data Availability

The primary data used to support the findings of this study are available from the corresponding author upon request.

## References

[1] Dutta P, Bhowmik S, Das S (2011). Methane and carbon dioxide sorption on a set of coals from India. Int. J. Coal Geol..

[2] Mathews JP, Chaffee AL (2012). The molecular representations of coal—A review. Fuel.

[3] Niekerk DV, Mathews JP (2010). Molecular representations of Permian-aged vitrinite-rich and inertinite-rich South African coals. Fuel.

[4] Song Y, Zhu Y-M, Li W (2017). Macromolecule simulation and CH4 adsorption mechanism of coal vitrinite. Appl. Surf. Sci..

[5] Li W, Liu H, Song X (2015). Multifractal analysis of Hg pore size distributions of tectonically deformed coals. Int. J. Coal Geol..

[6] Pan J (2016). Micro-pores and fractures of coals analysed by field emission scanning electron microscopy and fractal theory. Fuel.

[7] Zhang R (2015). Estimation and modeling of coal pore accessibility using small angle neutron scattering. Fuel.

[8] Firouzi M, Rupp EC, Liu CW, Wilcox J (2014). Molecular simulation and experimental characterization of the nanoporous structures of coal and gas shale. Int. J. Coal Geol..

[9] Zhao Y, Feng Y, Zhang X (2016). Molecular simulation of CO 2 /CH 4 self- and transport diffusion coefficients in coal. Fuel.

[10] Sarkisov L, Harrison A (2011). Computational structure characterisation tools in application to ordered and disordered porous materials. Mol. Simul..

[11] Anstine DM, Tang D, Sholl DS, Colina CM (2021). Adsorption space for microporous polymers with diverse adsorbate species. NPJ Comput. Mater..

[12] Pinheiro M, Martin RL, Rycroft CH, Haranczyk M (2013). High accuracy geometric analysis of crystalline porous materials. CrystEngComm.

[13] Willems TF, Rycroft CH, Kazi M, Meza JC, Haranczyk M (2012). Algorithms and tools for high-throughput geometry-based analysis of crystalline porous materials. Microporous Mesoporous Mater..

[14] Pinheiro M (2013). Characterization and comparison of pore landscapes in crystalline porous materials. J. Mol. Graph Model.

[15] Martin RL, Smit B, Haranczyk M (2012). Addressing challenges of identifying geometrically diverse sets of crystalline porous materials. J. Chem. Inf. Model.

[16] Martin RL, Haranczyk M (2014). Construction and characterization of structure models of crystalline porous polymers. Cryst. Growth Des..

[17] Priezjev NV, Makeev MA (2018). Structural transformations and mechanical properties of porous glasses under compressive loading. J. Non-Cryst. Solids.

[18] Brochard L, Vandamme M, Pellenq RJM (2012). Poromechanics of microporous media. J. Mech. Phys. Solids.

[19] Hong D, Liu L, Wang C, Si T, Guo X (2021). Construction of a coal char model and its combustion and gasification characteristics: Molecular dynamic simulations based on ReaxFF. Fuel.

[20] Kong X-P, Wang J (2016). Copper(II) adsorption on the kaolinite(001) surface: Insights from first-principles calculations and molecular dynamics simulations. Appl. Surf. Sci..

[21] Song Y, Jiang B, Li W (2017). Molecular simulation of CH4/CO2/H2O competitive adsorption on low rank coal vitrinite. Phys. Chem. Chem. Phys..

[22] Zhang J, Liu K, Clennell MB, Dewhurst DN, Pervukhina M (2015). Molecular simulation of CO2–CH4 competitive adsorption and induced coal swelling. Fuel.

[23] Langmuir I (1916). The constitution and fundamental properties of solids and liquids. Part I. solids. J. Am. Chem. Soc..

[24] Pajdak A, Kudasik M, Skoczylas N, Wierzbicki M, Teixeira Palla Braga L (2019). Studies on the competitive sorption of CO2 and CH4 on hard coal. Int. J. Greenhouse Gas Control.

[25] Brunauer S, Emmett PH, Teller E (1938). Adsorption of gases in multimolecular layers. J. Am. Chem. Soc..

[26] Dubinin MM, Stoeckli HF (1980). Homogeneous and heterogeneous micropore structures in carbonaceous adsorbents. J. Colloid Interface Sci..

[27] Hu B (2019). Effect of pulverization on the microporous and ultramicroporous structures of coal using low-pressure CO2 adsorption. Energy Fuels.

[28] Zhang J (2021). Molecular simulation of gases competitive adsorption in lignite and analysis of original CO desorption. Sci. Rep..

[29] Liu X, He X (2016). Effect of pore characteristics on coalbed methane adsorption in middle-high rank coals. Adsorption.

[30] Liu D (2021). An updated study on CH4 isothermal adsorption and isosteric adsorption heat behaviors of variable rank coals. J. Nat. Gas Sci. Eng..

[31] Yan J, Meng Z, Zhang K, Yao H, Hao H (2020). Pore distribution characteristics of various rank coals matrix and their influences on gas adsorption. J Petrol Sci Eng.

[32] Yin T, Liu D, Cai Y, Liu Z, Gutierrez M (2020). A new constructed macromolecule-pore structure of anthracite and its related gas adsorption: A molecular simulation study. Int. J. Coal Geol..

[33] Bai Y, Lin H-F, Li S-G, Yan M, Long H (2021). Molecular simulation of N2 and CO2 injection into a coal model containing adsorbed methane at different temperatures. Energy.

[34] Wen Z, Yang Y, Wang Q, Yao B, Xue Y (2021). Mechanism and characteristics of CH4/CO2/H2O adsorption in lignite molecules. Geofluids.

[35] Zhang D (2019). Influences of dynamic entrainer-blended supercritical CO2 fluid exposure on high-pressure methane adsorption on coals. J. Nat. Gas Sci. Eng..

[36] Wang J, Ding C, Gao D, Liu H (2020). Research on adsorption characteristics of H2S, CH4, N2 in coal based on Monte Carlo method. Sci Rep.

[37] Pan J, Hou Q, Ju Y, Bai H, Zhao Y (2012). Coalbed methane sorption related to coal deformation structures at different temperatures and pressures. Fuel.

[38] Mosher K, He J, Liu Y, Rupp E, Wilcox J (2013). Molecular simulation of methane adsorption in micro- and mesoporous carbons with applications to coal and gas shale systems. Int. J. Coal Geol..

[39] Liu Y, Zhu Y, Liu S, Li W, Tang X (2018). Temperature effect on gas adsorption capacity in different sized pores of coal: Experiment and numerical modeling. J. Petrol Sci. Eng..

[40] Wang Z (2018). Characteristics of microscopic pore structure and fractal dimension of bituminous coal by cyclic gas adsorption/desorption: An experimental study. Fuel.

[41] Nie B, Liu X, Yang L, Meng J, Li X (2015). Pore structure characterization of different rank coals using gas adsorption and scanning electron microscopy. Fuel.

[42] Chen S (2017). Pore structure characterization of different rank coals using N2 and CO2 adsorption and its effect on CH4 adsorption capacity: A case in panguan syncline, Western Guizhou, China. Energy Fuels.

[43] Zhao Y, Sun Y, Liu S, Chen Z, Yuan L (2018). Pore structure characterization of coal by synchrotron radiation nano-CT. Fuel.

[44] Liu H, Mou J, Cheng Y (2015). Impact of pore structure on gas adsorption and diffusion dynamics for long-flame coal. J. Nat. Gas Sci. Eng..

[45] Shan CA (2018). Effects of nano-pore system characteristics on CH4 adsorption capacity in anthracite. Front. Earth Sci..

[46] Yeganegi S, Gholampour F (2016). Simulation of methane adsorption and diffusion in a carbon nanotube channel. Chem. Eng. Sci..

[47] Liu X-Q (2016). Molecular simulation of CH4, CO2, H2O and N2 molecules adsorption on heterogeneous surface models of coal. Appl. Surf. Sci..

[48] Dang Y (2017). Molecular simulation of CO2/CH4 adsorption in brown coal: Effect of oxygen-, nitrogen-, and sulfur-containing functional groups. Appl. Surf. Sci..

[49] Guo X, He Y, Wang J (2021). Molecular structural characteristics of late Jurassic Shengli lignite submacerals. J. Solid State Chem..

[50] Wang J-P, Li G-Y, Guo R, Li A-Q, Liang Y-H (2016). Theoretical and experimental insight into coal structure: Establishing a chemical model for yuzhou lignite. Energy Fuels.

[51] Zhu H (2020). Methane adsorption influence and diffusion behavior of coking coal macromolecules under different moisture contents. Energy Fuels.

[52] Liu F-J (2014). Characterization of oxygen-containing species in methanolysis products of the extraction residue from xianfeng lignite with negative-ion electrospray ionization fourier transform ion cyclotron resonance mass spectrometry. Energy Fuels.

[53] Yoshida T, Maekawa Y (1987). Characterization of coal structure by CP/MAS carbon-13 NMR spectrometry. Fuel Process. Technol..

[54] Song C, Hou L, Saini AK, Hatcher PG, Schobert HH (1993). CPMAS 13C NMR and pyrolysis-GC-MS studies of structure and liquefaction reactions of Montana subbituminous coal. Fuel Process. Technol..

[55] Jinno K (1989). CP-MAS13C nuclear magnetic resonance spectra for identification of functionality of octadecylsilica bonded phases. J. Chromatogr. Sci..

[56] Franco DV, Gelan JM, Martens HJ, Vanderzande DJM (1991). Characterization by 13C CP/MAS n.m.r. spectroscopy of the structural changes in coals after chemical treatments. Fuel.

[57] Kawashima H, Takanohashi T (2001). Modification of model structures of upper freeport coal extracts using 13C NMR chemical shift calculations. Energy Fuels.

[58] Zhu H (2021). A Study on the Effect of coal metamorphism on the adsorption characteristics of a binary component system: CO2 and N2. ACS Omega.

[59] Vandenbroucke M, Largeau C (2007). Kerogen origin, evolution and structure. Org. Geochem..

[60] Gorbaty ML, Kelemen SR (2001). Characterization and reactivity of organically bound sulfur and nitrogen fossil fuels. Fuel Process. Technol..

[61] Grzybek T, Pietrzak R, Wachowska H (2002). X-ray photoelectron spectroscopy study of oxidized coals with different sulphur content. Fuel Process. Technol..

[62] Desimoni E, Casella GI, Morone A, Salvi AM (1990). XPS determination of oxygen-containing functional groups on carbon-fibre surfaces and the cleaning of these surfaces. Surf. Interface Anal..

[63] Gong B, Buckley AN, Lamb RN, Nelson PF (1999). XPS determination of the forms of nitrogen in coal pyrolysis chars. Surf. Interface Anal..

[64] Meng J, Zhong R, Li S, Yin F, Nie B (2018). Molecular model construction and study of gas adsorption of zhaozhuang coal. Energy Fuels.

[65] Jones AJ, Ostrouchov C, Haranczyk M, Iglesia E (2013). From rays to structures: Representation and selection of void structures in zeolites using stochastic methods. Microporous Mesoporous Mater..

[66] Düren T, Millange F, Férey G, Walton KS, Snurr RQ (2007). Calculating geometric surface areas as a characterization tool for metal−organic frameworks. J. Phys. Chem. C.

[67] Ongari D (2017). Accurate characterization of the pore volume in microporous crystalline materials. Langmuir.

[68] Gao D, Hong L, Wang J, Zheng D (2020). Molecular simulation of gas adsorption characteristics and diffusion in micropores of lignite. Fuel.

